# A Mixed‐Methods Evaluation of a Peer Group Intervention to Promote Wellbeing in Mental Health Nurses

**DOI:** 10.1111/inm.70032

**Published:** 2025-04-04

**Authors:** Alannah L. Cooper, Richard A. Read, Sally Burrows, Janie A. Brown

**Affiliations:** ^1^ School of Nursing, College of Health & Education Murdoch University Murdoch Western Australia Australia; ^2^ Royal Perth Bentley Group Centre for Wellbeing and Sustainable Practice Perth Western Australia Australia; ^3^ School of Medicine University of Western Australia Perth Western Australia Australia; ^4^ Royal Perth Research Foundation Perth Western Australia Australia; ^5^ School of Nursing Curtin University Bentley Western Australia Australia; ^6^ St John of God Midland Public and Private Hospital Midland Western Australia Australia; ^7^ The Western Australian Group for Evidence Informed Healthcare Practice (A JBI Centre of Excellence) Curtin University Bentley Australia

**Keywords:** intervention, mental health nurses, peer support, stress, wellbeing

## Abstract

The work mental health nurses undertake is widely acknowledged as being challenging. Stressors encountered in the workplace can negatively impact nurses' psychological wellbeing and contribute to issues with retaining nurses in the profession. There is limited interventional research that focuses on external factors that foster nurse wellbeing. This study aimed to evaluate a peer group intervention to promote nurse wellbeing. A longitudinal mixed‐methods study with an equal status concurrent design was undertaken. Qualitative and quantitative data were collected via semi‐structured interviews and surveys across three timepoints, baseline, mid‐intervention, and post‐intervention. Qualitative data were collected to explore interviewees' experiences of participating in the intervention, and quantitative data were obtained to assess for any measurable effect on wellbeing outcomes. Fifteen peer group participants completed semi‐structured interviews. There were *n* = 28 responses to the baseline survey, *n* = 27 returned the mid‐intervention survey, and *n* = 25 responded to the post‐intervention survey. Qualitative data analysis identified four main themes: Attending Peer Group, Participating in Peer Group, Impact on the Individual, and Unrelated Workplace Change. Wellbeing scores were found to be significantly modified by the number of peer group sessions attended for depression (*p* = 0.006), stress (*p* = 0.004), and emotional exhaustion (*p* = 0.02) By the post‐intervention survey, more favourable scores were significantly associated with higher attendance levels for all three measures. Integration of the qualitative findings and quantitative results demonstrated potential benefits of peer groups for nurse wellbeing. Given that greater exposure to the intervention was associated with better outcomes, facilitating attendance is essential to realise the benefits of peer groups.

## 1 Background

Mental health nurses (MHNs) face significant stressors in their work that can lead to negative impacts on their psychological wellbeing (Delgado et al. 2021; Foster et al. 2020). Stressors MHNs encounter include challenging behaviours from consumers, exposure to violence, inter‐and intra‐professional conflict, staffing shortages, poor skill mix, lack of resources and high acuity (Cranage and Foster 2022). These long‐standing stressors were further exacerbated by the coronavirus pandemic and have led to issues with retaining nurses in the profession (Adams et al. 2021; Buchan et al. 2022). Globally, nursing shortages are increasing (Buchan et al. 2022) making the provision of safe patient care increasingly challenging. Approaches to promote nurse wellbeing are urgently needed to protect nurses from harm, increase retention, and facilitate optimal patient care.

## 2 Introduction

In recognition of the challenging work environment MHNs face, ways to promote nurse wellbeing and resilience have begun to be investigated (Bui et al. 2023; Foster et al. 2021). Interventions have predominately targeted individuals and have often included education on strategies such as mindfulness (Bekelepi and Martin 2022; Foster et al. 2021). A systematic review of mindfulness‐based interventions in mental health nurses found slight improvements in psychological wellbeing and medium beneficial effects for psychological distress (Kang and Myung 2022). While these results indicate some benefit, interventions that focus primarily on what individuals can do are likely to be limited in their effectiveness (Bui et al. 2023; Cooper et al. 2022).

External factors are also known to impact nurse resilience and wellbeing (Bui et al. 2023; Cooper et al. 2022). The importance of the team within which nurses work and the ability of teams to provide peer support are external factors that influence nurse resilience and promote wellbeing (Cooper et al. 2020). Taking active steps to foster peer support and create supportive work environments could help nurses to collaborate and promote resilience and wellbeing. There has been little research focusing on developing and promoting peer support to improve MHNs experiences in the workplace (Bekelepi and Martin 2022; Bui et al. 2023). The aim of this study was to evaluate a peer group (PG) intervention to promote wellbeing in MHNs. The objectives were to:

1. Explore participants' experiences of participating in a PG intervention.
2. Identify enablers and barriers to PG participation,
3. Evaluate the effectiveness of the intervention on outcomes relating to nurse wellbeing.

## 3 Methods

### 3.1 Design

A longitudinal mixed‐methods study with an equal status convergent design (QUAL + QUAN) (Creswell and Creswell 2023; Nastasi et al. 2007) was conducted to evaluate a PG intervention for MHNs using the Transparent Reporting of Evaluations with Non‐randomised Designs (TREND) guidelines (Des Jarlais et al. 2004). Qualitative and quantitative data were collected concurrently over three timepoints. Qualitative data were collected to explore interviewees' experiences of participating in the intervention, and quantitative data were obtained to assess for any measurable effect on wellbeing outcomes. Integration mostly took place post data collection, at the interpretation and reporting level through merging where qualitative and quantitative data were drawn together (Fetters et al. 2013). At a method level, integration occurred via connecting through the sampling frame and embedding with quantitative data as a complementary data set to the qualitative findings (Creswell and Plano Clark 2018; Fetters et al. 2013).

### 3.2 Ethical Considerations

Ethical approval was sought and obtained from the study hospital Human Research Ethics Committee (RGS4393). Potential participants were provided with an information sheet that outlined the purpose and nature of the study and were given the opportunity to ask questions and discuss the project with the researchers. Support and counselling were available in the event a participant became distressed.

### 3.3 Study Setting

A public hospital in metropolitan Perth, Western Australia with 199 beds, of which 100 are inpatient mental health (MH) beds. The hospital provides MH services for young people and adults, comprising acute inpatient units, rehabilitation services, community services, and wellness clinics.

### 3.4 Participants

All enrolled and registered nurses working in MH services at the study site were invited to participate in the study via in‐person information sessions run by the research team. Potential participants were provided with a participant information sheet and had the opportunity to ask questions. All participants were requested to complete quantitative measures collected for the study, whereas taking part in the qualitative component via interviews was optional.

### 3.5 Intervention

The intervention consisted of 16 PG sessions (per participant) facilitated by the study hospital's wellbeing service between February and December 2022. The intervention aimed to promote wellbeing and to provide participants with a structured, supportive environment to share stressors and successes, and build collaborative, nurturing, and supportive relationships. The 60‐min sessions were run by two facilitators and drew on the foundations of Clinical Pastoral Education and utilised processes to develop self‐awareness through an action‐reflection approach (Clevenger et al. 2021). Confidentiality and trust within PGs drew on the principles of circles of trust (Palmer 2004) and a focus on core values, motivations, and behaviours mirrored those of values‐based reflective practice as described in detail by Bunniss (2021).

Initially, participants were allocated to a specific group with 6–7 participants per group, and the focus was on facilitated group reflective practice. Based on feedback received from the first eight sessions, modifications were made to allow nurses the flexibility to attend any session. Participants also expressed a desire for more structured sessions and for the topics to be predetermined by the facilitators. Content explored in the sessions included self‐awareness, burnout, resilience, personal core values, team formation and team building. The educational topics were used to initiate a facilitated group reflective discussion. Multimodal approaches were used to introduce topics and actively engage participants, including group discussions, motivational image cards, musical reflections, silent reflections, and brief formal presentations. During the PGs, the facilitators encouraged deep listening and reflection, pointed out responses that tried to fix the storyteller, and encouraged self‐reflection and insight in each participant. At the conclusion of each session, the facilitators met to identify/discuss any concerns about participant wellbeing. When concerns were identified, the relevant participant(s) were offered one‐to‐one follow‐up.

### 3.6 Data Collection

Data were collected on PG participant characteristics for the qualitative and quantitative components of the study. Qualitative and quantitative data collection occurred simultaneously across three timepoints: baseline (T1), mid‐way through the intervention (T2) and post‐intervention (T3). The qualitative data collected via interviews at T1 explored the stresses participants faced in their work. These baseline qualitative findings are fully presented in an earlier publication focusing on workplace context (Cooper et al. 2024). To address the aims and objectives of this paper, qualitative data from T2 and T3, where PG participants' experiences of the intervention were explored, are integrated with quantitative results from all timepoints to assess measurable effects on wellbeing outcomes.

#### 3.6.1 Interviews

Semi‐structured interviews to explore PG participants' experiences were conducted at T2 and T3. Open‐ended questions were developed by the researchers (Table 1). The semi‐structured interview style allowed the interviewer to follow up with additional questions to fully explore interviewees responses (Rubin 2012). The interviews were conducted by a female, PhD‐qualified, Nurse Researcher (AC) via telephone, at a time convenient to interviewees. To reduce the risk of bias, the Nurse Researcher was independent of the design and delivery of the intervention and had no prior relationship with the interviewees. Informed written consent was obtained from interviewees prior to the interviews. The interviews were audio recorded and transcribed verbatim.

**TABLE 1:** Interview questions.

How is your participation in the study going? (PROMPT: ask about attendance and if there have been difficulties with attending ask about reasons/barriers)
What have been the most helpful aspects of the peer groups for you? (PROMPT: ask about personal and professional aspects)
Are there any aspects of the peer groups that have made things more difficult for you? (PROMPT: ask about personal and professional aspects)
Have you changed any practices in your life as a result of the peer groups? (PROMPT: ask about personal and professional aspects)
Are there things you would have liked to change but were unable to? (PROMPT: ask about personal and professional aspects)
Do you have plans for doing things differently in the future as a result of the peer groups? (PROMPT: ask about personal and professional aspects)
We are nearly finished, do you have anything else you would like to add about the impact of the peer groups on how stressed you feel as a result of your work?

#### 3.6.2 Wellbeing Related Outcomes

To assess for any measurable effect of the intervention data on wellbeing and related outcomes of depression, anxiety, stress, resilience, burnout, and quality of life were collected at T1, T2 and T3. The five validated instruments used are outlined in Table 2 (Campbell‐Sills and Stein 2007; Dyrbye et al. 2016; Lovibond and Lovibond 1995a; Maslach et al. 1996; Skevington et al. 2013).

**TABLE 2:** Summary of instruments administered to measure wellbeing‐related outcomes.

Instrument	Purpose	Number of items	Scoring and reliability
Abbreviated 10‐item Connor‐Davidson Resilience Scale (CD‐RISC10) Campbell‐Sills and Stein (2007)	Measures participants' resilience levels	10 items	Higher scores indicate higher levels of resilience. Cronbach's alpha 0.85 Campbell‐Sills and Stein (2007)
Depression, Anxiety and Stress Scale (DASS) Lovibond and Lovibond (1995a)	Assesses mental health	21 items	Lower scores are indicative of lower levels of depression, anxiety, and stress. Cronbach's alpha 0.93 Henry and Crawford (2005)
Maslach Burnout Inventory Human Services Survey for Medical Personnel (MBI) Maslach et al. (1996)	Measures three dimensions of burnout; Emotional Exhaustion, Depersonalisation, and Personal Accomplishment	22 items	Higher scores for Emotional Exhaustion and Depersonalisation indicate greater levels of burnout whereas lower scores for Personal Accomplishment indicate a higher level of burnout. Cronbach's alpha for the three dimensions ranges from 0.76 to 0.9 Kalliath et al. (2000)
Well‐Being Index (WBI) Dyrbye et al. (2016)	Assesses summative wellbeing	9 items	Higher scores indicate a greater degree of distress, lower levels of satisfaction with work‐life balance, and lower meaning in work
WHOQOL‐SPRB BREF Skevington et al. (2013)	Measures spiritual, religious, and personal beliefs within quality of life	9 items	Higher scores indicate a greater level of spiritual wellbeing. Cronbach's alpha of 0.85 Skevington et al. (2013)

### 3.7 Analysis

#### 3.7.1 Qualitative

Using reflexive thematic analysis following the six iterative steps outlined by Braun and Clarke (2006) presented in Table 3, patterns of shared meaning were identified in the qualitative data, and themes were developed (Braun and Clarke 2019).

**TABLE 3:** Steps of reflexive thematic analysis process (Braun and Clarke 2006).

Phase	Actions
Familiarising yourself with the data	Audio recordings listened to multiple times and transcriptions of recordings were made
Generating initial codes	Transcripts read in entirety multiple times. Initial codes independently generated (AC & JB). NVivo was used to organise data and record codes
Searching for themes	Searched for themes by assessing for similarity of concepts within the generated codes (AC & JB)
Reviewing themes	Themes reviewed and discussed by the research team
Defining and naming themes	Refinement of the specifics of each theme, theme names finalised, and theme definitions generated
Producing the report	Themes written with supporting evidence within the data and findings written up

#### 3.7.2 Quantitative

Data are summarised as mean and SD, or count and percentage as appropriate. Due to the bounded nature of the scores examined, fractional logistic regression was employed to investigate change over time in the scores. Scores were converted to a 0 to 1 scale prior to analysis [(score—min possible score)/(max possible score—min possible score)]. Each participant's set of responses over time was treated as a cluster using Stata's vce cluster option. Models were adjusted for potential age and gender effects as well as possible PG session effects. The interaction of time and the number of sessions was also tested. Mean scores on untransformed scales are provided by categories of the number of sessions; however, the actual number of sessions was used in the regression analysis. Analyses were performed using Stata 17 (StataCorp 2021) and alpha was set at 0.05.

#### 3.7.3 Integration

To facilitate side‐by‐side comparison and draw meta inferences from the data, qualitative findings and quantitative results were integrated using a joint display (Haynes‐Brown and Fetters 2021). The fit of integration was assessed by examining the data sources for confirmation, expansion, and discordance (Fetters et al. 2013). Confirmation refers to when findings from data sources confirm each other, expansion is when the findings from data sources broaden insights, and discordance is when the findings from the data sources are inconsistent, contradict, or disagree with each other (Fetters et al. 2013).

### 3.8 Qualitative Findings

**FIGURE 1:**
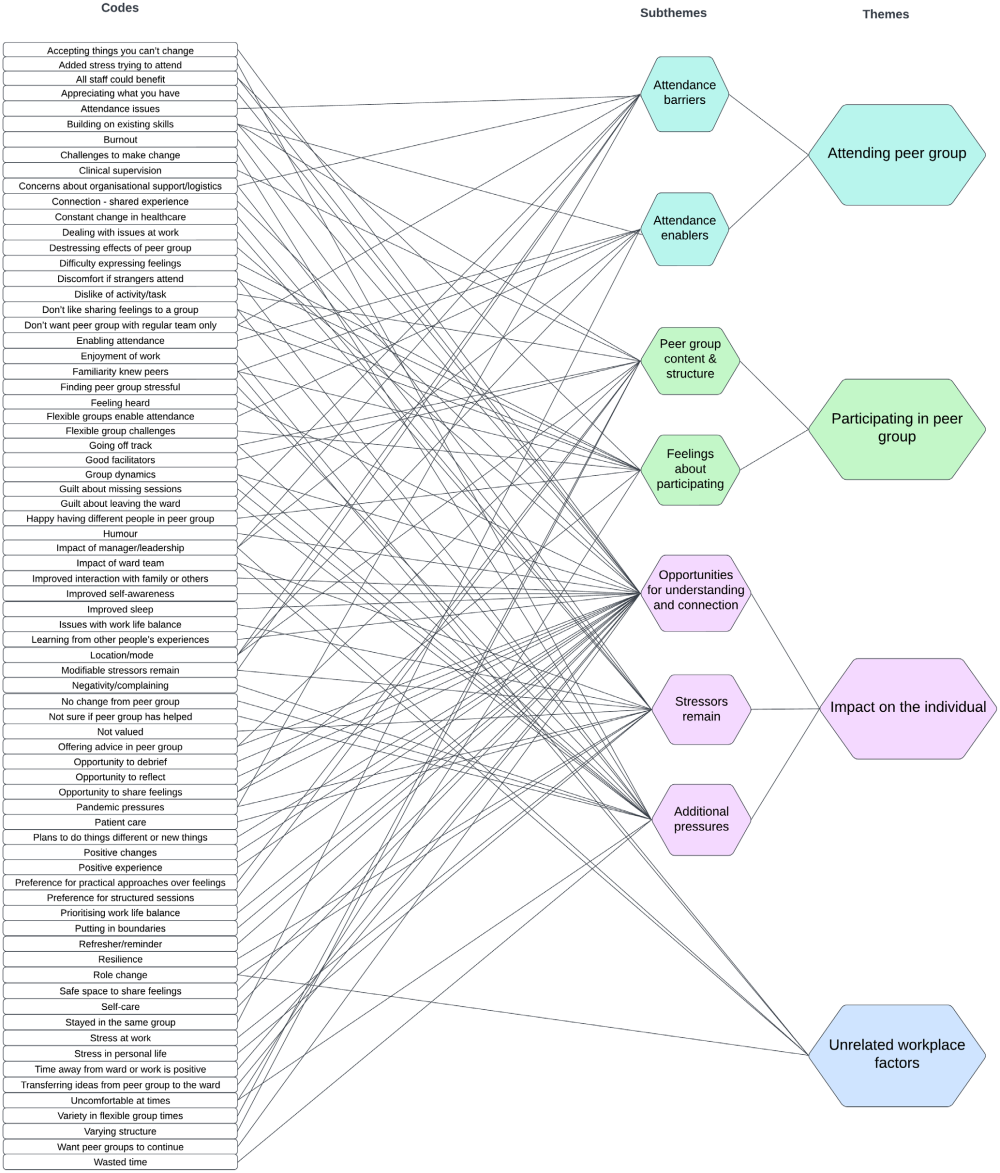
Thematic map.

A total of 15 MHNs completed T2 and T3 interviews. Purposive sampling was used to include nurses with a range of experience and those with low, medium, and high rates of PG attendance (Table 4). The T2 interviews were conducted mid‐intervention between June and August 2022. The duration ranged from 8 to 31 min. Final T3 interviews were conducted in December 2022 after the last PG sessions and the duration ranged from 8 to 26 min. Four main themes and seven subthemes were derived from 74 codes generated in the analysis of the interviews (Figure 1). The four main themes were Attending Peer Group, Participating in Peer Group, Impact on the Individual and Unrelated Workplace Change.

**TABLE 4:** Interviewee characteristics.

Characteristic	Sample (*n* = 15)
Mean age (range)^a^	41 (24–63) years
Female	53% (*n* = 8)
Registered Nurse	93% (*n* = 14)
Enrolled Nurse	7% (*n* = 1)
Part‐time	27% (*n* = 4)
Full‐time	73% (*n* = 11)
Mean years of nursing experience	15 (2–41 years)
Mean years mental health experience	14 (1–41 years)
Peer group attendance	
Low attendance < 50%	33.3% (*n* = 5)
Medium attendance ≥ 50%–< 74%	33.3% (*n* = 5)
High attendance ≥ 75%	33.3% (*n* = 5)

^a^ One interviewee chose not to disclose their age.

### 3.9 Theme 1: Attending Peer Group

Practical issues with attending PG featured strongly in the T2 and T3 interviews. Barriers and enablers to attendance were discussed by interviewees.

#### 3.9.1 Attendance Barriers

The main barrier to attending sessions was the clinical demands of interviewees work. The busyness of wards and the need to prioritise patient care were frequently raised; ‘There are other priorities that you have to take into account. You can't just jump ship when there's things that need to be addressed in the ward’ (P3, T2). This was a source of frustration as interviewees wanted to attend PGs; ‘…it's not due to me not wanting to attend, it's just literally I can't get out’ (P16, T2). Some had issues with not being rostered on when their PG was scheduled ‘I haven't managed to go to a lot of the sessions because my roster hasn't facilitated it’ (P20, T2).

In the context of a busy work environment with competing demands and stresses, forgetting about PGs was an issue ‘…the only problem is when it slips your mind…’ (P26, T2). Issues with attendance were noted at the T2 interviews. Those able to attend recognised that other demands prevented their colleagues attending and resulted in very small groups; ‘There's not really many that attend… that's just because they've not been able to facilitate the time to actually sit there and be in the group’ (P14, T2). Reflecting on PG attendance at T3, the exacerbation of staffing shortages and difficulties with workload in the context of the pandemic were an additional barrier to attendance; ‘…because of our workload… at that time there was COVID… people were going off sick… staff were being pulled back to the wards…’ (P30, T3). These descriptions highlight the busy and complex work environment participants encountered and the difficulties of leaving their clinical work to attend PG.

#### 3.9.2 Attendance Enablers

Working with a supportive Nurse Manager (NM) was an important factor that could enable attendance; ‘My manager is quite good. She's always allocated time for us to go…’ (P1, T2). Active facilitation by NMs allowed nurses to leave busy clinical environments however, attending PG could cause difficulties; ‘I was surprised how willing the NUM's are at letting you go… given that sometimes attending them was inconvenient to the ward’ (P8, T3). Some interviewees working in community settings could plan and diarise attendance around client visits; ‘I've just scheduled it in my diary to make sure my clients are aware I'm not available at that time’ (P14, T2). Having the flexibility to hold PGs online also enabled attendance; ‘I've been trying to get to every meeting. I think twice we did it online’ (P22, T2).

Adaptations after T2 that allowed PG participants to attend any session boosted numbers; ‘…when we connected as two groups at least there was a lot of us… it took off some of that stress because I was getting stressed going to them’ (P14, T3). Based on the feedback received during the T2 interviews reminders were sent about upcoming sessions and interviewees reported that receiving these, along with increased flexibility aided attendance; ‘Having email and texts to remind people … having the availability to change if you needed because of work commitments… were much better’ (P25, T3). Overall, participants reported that flexible attendance and reminders were valuable in the context of busy and unpredictable clinical work environments.

### 3.10 Theme 2: Participating in Peer Group

Discussions around PG participation revealed two key areas: the content and structure of the sessions, and how interviewees felt about taking part in PGs.

#### 3.10.1 Peer Group Content and Structure

The role of the facilitators conducting the PGs was a positive feature. Interviewees discussed how the facilitators supported them; ‘The staff running it are very helpful. They give you time to talk and they listen to you’ (P9, T2). There was wide agreement that the facilitators were accessible and easy to talk to; ‘…[the facilitators] they're very approachable people’ (P3, T3). There were mixed responses about the multimodal approaches used to introduce and explore topics during PGs, with personal preferences and learning styles raised; ‘…they liked the cards, don't they? (Laughs). To stimulate discussion… I don't find that helpful’ (P26, T3). Another interviewee enjoyed the prompting; ‘I liked the cards… particularly the last one that we did with the storytelling…’ (P29, T3).

A desire to get straight into discussions was expressed, reflecting busy clinical work environments and interviewees wanting to do something they perceived to be meaningful or tangible; ‘I didn't think much of the music (laughs) at the start. Just get into it and get on with it’ (P29, T3). The importance of keeping on track was also raised:

…some… peer group members are quite tangential and disorganised in terms of presenting their thoughts… we don't really get anywhere… I do find there is value to the groups… but I just don't think it has been as effective as it could be. (P16 T2)

Interviewees also expressed a strong preference for a structured approach, wanting a specific topic or material that they could engage with:

We have been more focused this time… which I found good, instead of just talking about this and that… I think that's a pointless exercise. But if we have something to bite our teeth into, I think that's the way it should be. (P7, T2)

The structured approach helped to keep the focus on supporting wellbeing and building collaborative relationships; ‘…when it was kind of a free for all, it just became very negative… when it was structured, we were coming back to positives…’ (P14, T3). The more structured approach to PG was perceived to be more beneficial in participants' descriptions.

#### 3.10.2 Feelings About Participating

The PGs made interviewees feel heard and valued; ‘…it feels as if someone cares, someone is listening to you’ (P9, T2), although some interviewees had difficulty expressing their feelings. This was attributed by one interviewee as being a personal trait rather than specific to PG; ‘…any time I hear the word feeling I shut off’ (P14, T2). Another interviewee described how they felt comfortable when focusing on work‐related issues or feelings but not their personal life. Interestingly, while this interviewee chose not to discuss their personal life within PG, they valued the opportunity to share with the facilitators in one‐to‐one follow ups; ‘…I felt safe enough to disclose whatever I needed… in front of facilitators, and I feel safest when I am by myself with them…’ (P22, T2).

The flexibility to attend any session was well received. Due to their familiarity with nursing staff across the hospital, many interviewees expressed that they felt comfortable with having different people attend sessions; ‘it wasn't much difference… I knew most of them… they're not strangers for me, except a few but it wasn't too difficult’ (P22, T3). One interviewee described feeling hesitant about having new attendees at first:

…you just feel a bit of… distrust because you've been in that small group for so long and they know you very well and you've built that bond… going to another group… I think it took two peer groups just to be just like, “It's alright… just go for it.” (P1, T3)

Some interviewees expressed a preference for the exposure to different people that the flexible attendance created, and felt connecting with more people made the sessions more stimulating; ‘I think it was better than having the same people, it was good to go on different days to have different people because people interact very differently… it was really good when I went to different sessions’ (P25, T3). Overall, flexible attendance was acceptable to participants.

### 3.11 Theme 3: Impact on the Individual

Overall, the intervention was positively received, and interviewees described a wide range of benefits from attending, particularly in relation to having the opportunity to connect with others and to further their understanding of self. There was acknowledgement that although PGs were helpful, stressors inside and outside the workplace remained. Some interviewees also discussed how attending PG could at times be perceived as an additional pressure.

#### 3.11.1 Opportunities for Connection and Understanding

There was widespread agreement amongst interviewees that attending PGs had been beneficial. The opportunity to connect with others during sessions was valuable and made interviewees feel less alone; ‘…it's good actually, knowing that others are feeling similarly to you…’ (P26, T2). For some interviewees, hearing other people's life experiences helped put their own issues into perspective; ‘I did find it helpful to be reminded of other people's struggles just in… a sense of taking… how lucky I am in so many ways… I was so consumed with being stressed out at work’ (P20, T3). Peer groups provided an opportunity for connection; ‘I think the peer groups are important… it's a venue to share ideas… when you get a few brains gathered together with the same focus, that's always helpful and we don't get that at work…’ (P7, T2).

By creating a safe space to share experiences and feelings, PGs helped participants manage stress; ‘…it helps with stress levels and a chance to ventilate… without any judgement…’ (P8, T2). Some interviewees discussed how the sessions provided an opportunity to decompress and not take work stress home; ‘…when I go home on the days I have peer group, I've already ventilated my feelings, I feel unwound… I feel lighter before I go home…’ (P1, T2). Being encouraged to take time to reflect improved relationships with self and others; ‘I think I'm more patient with myself… I give more kindness to myself… that sort of trickles into my work I'm more patient and kinder to other people…’ (P16, T3). Some interviewees also reported that the sessions re‐emphasised the importance of self‐care and some made efforts to improve their health through exercise, sleep hygiene, making time to socialise, and reassessing their work‐life balance.

Discussions in PGs gave interviewees an opportunity to gain a greater understanding of their behaviours; ‘Some of the topics that were brought up I found useful… I could put them into perspective, from the things I would normally do at work and recognise where those things came from’ (P8, T3). The increased self‐awareness interviewees reported was something they thought would benefit other staff and the teams they worked within ‘…if most of the staff could get that experience and have those sessions, that would be very productive… I feel I have a different knowledge or awareness level…’ (P12, T2). Most interviewees expressed that they would like PG to be available to all staff, although there was acknowledgement that facilitating attendance would be difficult.

#### 3.11.2 Stressors Remain

While PG had a range of benefits, interviewees were aware that the intervention did not remove or change stressors. These included ongoing issues with staffing levels and skill mix, that created unmanageable workloads; ‘After one shift you feel so tired because of the mental energy you put in… you're carrying sometimes two or three people's workload.’ (P7, T2). The impacts of a stressful work environment left interviewees drained and could make it difficult to engage with PG; ‘I… skipped out on some of the sessions because I was completely exhausted and at burnout’ (P20, T2). The pandemic exacerbated pre‐existing issues interviewees faced in their work; ‘…people being pulled away for COVID leave… really amplified all the problems that we've had…’ (P29, T3).

Some interviewees reported difficulty with managers, that worsened a challenging work environment; ‘…there are some managers who are quite… rude and nasty…’ (P12, T3). Not feeling valued and the current state of healthcare were significant stressors; ‘…it's been a hard year… we've all felt really underappreciated and under supported by the executives… I'm… at the beginning of the realisation… the shock of the horror that is healthcare has arrived…’ (P20, T3). Issues with work‐life balance and difficulties disconnecting from work persisted for some; ‘…I had some days off sick… I'm still checking my emails’ (P29, T3). In addition to significant adversity in the workplace many interviewees also discussed sources of stress in their personal lives ‘I've dealt with both my parents passing away… and now I'm dealing with cancer…’ (P3, T2). Participants were aware how external sources of stress in their personal lives could impact on them in the workplace.

#### 3.11.3 Additional Pressures

Some interviewees described negative impacts that PGs could have, especially in the context of busy clinical work environments; ‘…feels like a little bit wasted time sometimes… your mind goes to thinking, “Oh god, I've got this care plan to write, I've got this plan to do”…’ (P25 T3). Conversely, some interviewees who were unable to attend PG, worried that they were failing to contribute; ‘…I do feel bad, because it is something I signed myself up to do and I feel like I'm letting the group down when I can't be there’ (P12, T2). Peer groups could also be an additional form of emotional labour when taking on other people's concerns and issues; ‘I was really tired, it was at the end of my shift… someone… was – not hogging the conversation – but it was very centred around what she needed to talk about, which was fine, but I was so tired…’ (P20, T2).

A lack of engagement during PGs could lead to awkward moments; ‘…it's horrible when the topic is put out… and no one talks and there's this uncomfortable silence’ (P30, T3). Additional tasks such as journalling were unrealistic for some interviewees; ‘I was thinking of doing a journal, I know (facilitator) told us to keep a little journal for six weeks but when I go home, the kids, me, I just don't have the time’ (P1, T2). One interviewee described how they were unsure about the impact of PG; ‘I don't know whether I've benefited… sometimes it has just been nice to use that as an excuse to get away from the ward… but at other times, it has felt like a bit of a… pressure to go’ (P20, T3). These descriptions show the mixed experiences participants had when trying to leave demanding and busy clinical work environments to attend PG.

### 3.12 Theme 4: Unrelated Workplace Change

The impact of changes in the workplace unrelated to PG was frequently raised by interviewees. The opening of the Western Australian borders and the subsequent spread of COVID were significant stressors that coincided with the intervention. Additional pressures related to COVID compounded existing stressors interviewees already faced in their work:

‘…at the moment things are settling and all those misunderstandings and all of those things that happened in the past is cooling off, and it's to do with COVID, I can understand that we are always stressed and not knowing what's happening… all this uncertainty was making us all really stressed’ (P22, T2)

One interviewee described how moving to a different ward with a Nurse Manager they perceived to be a better leader had a positive impact; ‘…I've become more assertive, but then I suppose the manager helps as well, because I've got a manager who is receptive’ (P7, T3). Another interviewee described how a role change that involved taking on more responsibility had increased their stress levels during the intervention period, however they felt that PGs had helped with taking on these new challenges; ‘it's hard to determine whether my stress levels are better managed… because the level of stress is increased due to the nature of the work. But I feel like I've been enabled with strategies to manage my stress better’ (P16, T3). This participant's reflection illustrates how PG had helped them navigate the transition to their new role.

## 4 Quantitative Results

Initially 32 MHNs enrolled into the PGs. Of these, *n* = 28 submitted baseline quantitative data at T1, *n* = 27 returned the T2 survey, and *n* = 25 responded to the T3 survey. The surveys were distributed in January 2022 for T1, June 2022 for T2, and December 2022 for T3. One participant withdrew before T2 due to a change in employment, and two participants were lost to follow up at T3. Respondent characteristics are presented in Table 5.

**TABLE 5:** Survey respondent characteristics.

Characteristic	T1 (*n* = 28)	T2 (*n* = 27)	T3 (*n* = 25)
Mean age (range)^a^	45 (23–62)	44 (23–62)	43 (23–62)
Female	64% (*n* = 18)	59% (*n* = 16)	60% (*n* = 15)
Registered Nurse	89% (*n* = 25)	93% (*n* = 25)	92% (*n* = 23)
Enrolled Nurse	11% (*n* = 3)	7% (*n* = 2)	8% (*n* = 2)
Part‐time	36% (*n* = 10)	41% (*n* = 11)	44% (*n* = 11)
Full‐time	64% (*n* = 18)	59% (*n* = 16)	56% (*n* = 14)
Mean years of nursing experience	13 (< 1–41 years)	13 (< 1–41 years)	13 (< 1–41 years)
Mean years mental health experience	12 (< 1–41 years)	11 (< 1–41 years)	11 (< 1–41 years)

^a^ Two respondents chose not to disclose their age at T1, and one respondent did not disclose their age at T2 and T3.

### 4.1 Wellbeing‐Related Outcomes

As shown in Table 6, mean scores for stress and depersonalisation changed significantly over time (*p* = 0.04 and 0.03 respectively). Mean scores for stress increased from T2 to T3, indicating mean stress levels increased. While the increase in stress levels was statistically significant, levels remained in the normal clinical range across all time points (Lovibond and Lovibond 1995b). Mean scores for depersonalisation increased across the three time points. Clinically, the mean depersonalisation scores suggest low burnout at T1 and moderate burnout at T2 and T3 (Maslach et al. 1996). No statistically significant changes were detected over time for the other outcomes.

**TABLE 6:** Change in scores over time.

	Time 1		Time 2		Time 3		Unadjusted	Adjusted^b^
	*N*	Mean (SD)	*N*	Mean (SD)	*N*	Mean (SD)	*p* ^a^	*p* ^a^
Depression	25	2.5 (2.7)	25	2.0 (2.2)	23	2.9 (2.7)	0.15	0.15
Anxiety	25	3.7 (3.4)	25	2.9 (2.6)	23	3.4 (2.7)	0.38	0.3
Stress	25	3.7 (3.4)	26	3.3 (2.4)	23	4.3 (3.2)	0.051	0.04^c^
Resilience	25	30.6 (5.7)	26	29.8 (6.0)	23	30.0 (5.6)	0.79	0.97
Emotional exhaustion	25	21.1 (9.1)	26	19.3 (11.8)	23	18.9 (14.2)	0.6	0.46
Depersonalisation	25	3.9 (3.4)	26	5.5 (5.6)	23	6.1 (6.1)	0.07	0.03^d^
Personal accomplishment	25	36.1 (7.0)	26	34.3 (6.3)	23	33.8 (7.6)	0.38	0.28
Wellbeing	21	1.5 (2.3)	26	1.7 (2.7)	23	1.2 (2.9)	0.58	0.76
Spiritual wellbeing	25	30.1 (8.5)	26	29.0 (8.3)	23	28.4 (9.0)	0.5	0.71

^a^ *p* values from fractional logistic regression of scores converted to 0 to 1 scale. Summary statistics are on untransformed scores.

^b^ Adjusted for age, gender, and the number of PG sessions attended.

^c^ Significant change over time between T2 and T3.

^d^ Significant change over time from T1 to T2 and T1 to T3.

Over time, wellbeing scores were found to be significantly modified by the level of exposure (number of sessions attended) for depression, stress, and emotional exhaustion (Table 7), and the interaction of time and number of sessions (*p* values 0.006, 0.004 and 0.02 respectively). By T3, more favourable scores were significantly associated with higher attendance levels for all three measures. Clinically, mean depression and stress levels were in the normal range for all participants, while mean exhaustion levels indicated moderate burnout at lower attendance levels (Lovibond and Lovibond 1995b; Maslach et al. 1996).

**TABLE 7:** Change over time in scores by number of sessions attended.

	Sessions^b^	Time 1		Time 2		Time 3		Interaction
		*N*	Mean (SD)	*N*	Mean (SD)	*N*	Mean (SD)	*p* ^a^
Depression	≤ 5	8	2.9 (2.1)	8	1.3 (1.3)	7	4 (2.1)	
	6–10	9	1 (1.5)	9	1.8 (1.6)	8	2.3 (2.6)	
	11–16	8	3.9 (3.6)	8	3 (3.3)	8	2.6 (3.2)	0.006
Anxiety	≤ 5	8	4.4 (3.1)	8	3.3 (3.3)	7	4.4 (3.0)	
	6–10	9	2.7 (1.9)	9	2.4 (2.2)	8	3.1 (2.3)	
	11–16	8	4.1 (4.9)	8	3.1 (2.3)	8	2.8 (3.0)	0.06
Stress	≤ 5	8	4.5 (3.8)	9	3.6 (2.4)	7	6.4 (3.1)	
	6–10	9	2.2 (1.5)	9	2.9 (1.8)	8	3.8 (2.9)	
	11–16	8	4.6 (4.1)	8	3.4 (3.2)	8	2.9 (2.9)	0.004
Resilience	≤ 5	8	30.9 (3.6)	9	30.4 (6.7)	7	30.6 (5.1)	
	6–10	9	31.8 (6.3)	9	28.2 (6)	8	28.5 (6.8)	
	11–16	8	28.9 (7)	8	31 (5.5)	8	31.1 (5.0)	0.43
Emotional exhaustion	≤ 5	8	20.6 (7)	9	18.3 (11.7)	7	23.6 (16.1)	
	6–10	9	19.7 (10.9)	9	21.9 (12.7)	8	18.9 (12.3)	
	11–16	8	23.3 (9.5)	8	17.5 (11.8)	8	14.9 (14.8)	0.02
Depersonalisation	≤ 5	8	5.6 (4.4)	9	8.7 (6.9)	7	9.7 (7.5)	
	6–10	9	3.3 (3.1)	9	3.3 (4.6)	8	4.6 (4.6)	
	11–16	8	2.8 (2.2)	8	4.4 (3.6)	8	4.5 (5.3)	0.57
Personal accomplishment	≤ 5	8	33.6 (8.8)	9	32 (7.3)	7	30.7 (8.4)	
	6–10	9	37.2 (5.7)	9	33.3 (5.6)	8	33.4 (7.9)	
	11–16	8	37.4 (6.4)	8	38 (4.4)	8	36.9 (6.1)	0.11

^a^ *p* values from fractional logistic regression of scores converted to 0 to 1 scale. Summary statistics are on untransformed scores.

^b^ Number of sessions provided in categories for summary statistics but continuous form was used in regression.

### 4.2 Integration

Qualitative findings and quantitative results were drawn together to identify mixed methods meta‐inferences with reference to the study objectives (Teddlie and Tashakkori 2009). Completion of the joint display identified three areas of confirmation, two areas of expansion, and one area of discordance between the qualitative findings and quantitative results (Data S1). Quantitative results that demonstrated a measurable improvement in attendance levels after T2 confirmed participants reports in the qualitative data that increased flexibility had better facilitated their PG attendance. The qualitative findings expanded on the measurable improvement in attendance, with participants describing the factors that assisted and impeded their ability to attend PG. For example, some participants reported that receiving reminders about upcoming PGs helped boost their attendance.

In relation to nurse wellbeing, the quantitative results of increased stress and burnout levels confirmed participants accounts of pre‐existing workplace stressors being worsened in the context of the COVID‐19 pandemic. Although stress and burnout levels increased, they remained within clinically normal ranges, confirming participants descriptions that PG was generally perceived as beneficial. Quantitative results expanded on participants perception that PG could be beneficial by demonstrating that higher levels of exposure to the intervention were associated with better outcomes. However, there was also discordance between the qualitative findings and quantitative results, with some participants describing how trying to attend PG could sometimes be an additional stressor.

## 5 Discussion

The aim of this study was to evaluate a PG intervention to promote wellbeing in MHNs. While the intervention was widely viewed as beneficial, underlying issues with workplace conditions were a barrier to attendance. Greater exposure to the intervention was associated with better outcomes, highlighting the importance of employers and managers facilitating MHNs attendance. The intervention was positively received by participants, with many reporting a desire to continue to have PGs in the future. Feedback from participants demonstrated that PGs promoted social support in the workplace, a known attribute of nurse resilience (Cooper et al. 2020) and nurse wellbeing (Xiao et al. 2022). Creating a dedicated time for MHNs to come together fostered connection and opportunities to share experiences. This in turn made participants feel valued and heard, which is essential to enabling nurses to practice effectively (Kowalski et al. 2020). This is particularly important in the context of the isolation that nurses can feel in their work (Cranage and Foster 2022; Diaw et al. 2020).

The barriers to PG attendance were reflective of issues reported in the literature of suboptimal work conditions MHNs experience, including staffing shortages, poor skill mix and high patient acuity (Cranage and Foster 2022). Modifications during the intervention period to make attendance flexible boosted participation and allowed for the unpredictable nature of the clinical environment. The importance of supportive nurse managers in facilitating attendance was also highlighted, emphasising the key role nurse managers have in supporting nurse wellbeing (Niinihuhta and Häggman‐Laitila 2022). Nurse managers who promote healthy work cultures can positively influence nurse retention (Cardiff et al. 2023).

Similarly, to Foster et al.'s (2018) feasibility study of a resilience programme in MHNs, the intervention in this study was generally well received however, participants recognised that the intervention did not address modifiable workplace conditions. These findings highlight the need for broader interventions to address workplace conditions alongside interventions to provide support to nurses. This is particularly important as the perception, in this study, that the intervention could be an additional stressor was due to issues with staff shortages, participants not being able to leave their area of work due to clinical demands or feeling so drained from their work that it was difficult to engage.

Given the context of the opening of the state borders prior to T2, exposure to COVID and the implications this had for health services coinciding with the intervention, the increase in stress levels observed at T2 and T3 was unsurprising. The quantitative results were consistent with qualitative findings, where participants discussed ongoing workplace stressors and the additional pressures of the pandemic. Stress levels remaining within a clinically normal range (Lovibond and Lovibond 1995b) despite an exceptionally stressful time suggest that the social support fostered through the intervention assisted participants. Expanding on the benefits participants described from the intervention, the quantitative results showed higher levels of attendance were associated with improved outcomes for depression, stress, and emotional exhaustion, demonstrating that facilitating attendance is crucial to optimising the potential effects of the intervention. However, issues with PG attendance and assessing for significant changes to measures that remained within a clinically normal range are likely to have limited the detection of potential benefits that may have resulted from the intervention.

### 5.1 Limitations

This study was limited to a small group of MHNs at one hospital, which may affect the generalisability of the findings to other settings. The quantitative results must be interpreted with caution as the small sample size increases the potential risk of a Type 1 error (Knudson and Lindsey 2014). The authors also acknowledge that the type of person that was able to attend PG more frequently may generally be more likely to benefit from PG, or their life may be less complicated as opposed to greater participation being the only contributing factor for the differences observed. While the effect of improved wellbeing scores was not found in all measures examined, statistically significant improvements in three measures would suggest there is likely to be some merit in the association between increased attendance and improved outcomes for depression, stress, and emotional exhaustion. The characteristics and attitudes of participants who volunteer to take part in research may differ from individuals who choose not to participate; therefore, the sample may not be representative of MHNs.

## 6 Conclusion

In this study we evaluated a PG intervention to promote wellbeing in MHNs. The intervention was acceptable to participants, with qualitative findings and quantitative results demonstrating potential benefits of attending PGs for MHN wellbeing. Given that greater exposure to the intervention was associated with better outcomes, facilitating MHNs attendance is paramount, and issues with workplace conditions that prevent attendance need to be addressed by organisations.

## 7 Relevance for Clinical Practice

The work MHNs undertake is challenging and can have negative impacts on psychological wellbeing (Delgado et al. 2021; Foster et al. 2020) therefore, finding ways to support MHNs and promote wellbeing is essential. Peer Group is a pragmatic intervention that can be implemented in clinical practice to support nurse wellbeing.

## Author Contributions

Each named author has substantially contributed to conducting the underlying research and developing or reviewing this manuscript. All authors are in agreement with the manuscript submitted.

## Conflicts of Interest

The authors declare no conflicts of interest.

## Supporting information


Data S1

## Data Availability

The data that support the findings of this study are available on request from the corresponding author. The data are not publicly available due to privacy or ethical restrictions.
