# Supplementary material for: A Mixed‐Methods Evaluation of a Peer Group Intervention to Promote Wellbeing in Mental Health Nurses
Source: Int J Ment Health Nurs. 2025 Apr 4;34(2):e70032. doi: 10.1111/inm.70032 (PMC11969422; doi:10.1111/inm.70032)
Supplement: Supplementary file 1 — Data S1 [file INM-34-0-s001.docx]

**Joint display**

| **Study objective** | **Qualitative findings** | **Quantitative results** | **Mixed methods meta-inferences** |
| --- | --- | --- | --- |
| Explore participants’ experiences of participating in a peer group intervention | Subtheme: Peer Group Content and Structure:   - Facilitators were approachable, - Mixed responses to multi modal approaches, - Preference for a structured approach.   Subtheme: Feelings about Participating:   - Felt heard and valued, - Good to share experiences.   Subtheme: Stressors Remain   - Could be difficult to engage in peer group sometimes. |  |  |
| Identify enablers and barriers to peer group participation | Subtheme: Attendance Enablers   - Increased flexibility to attend any session help attendance, - Email and text reminders were helpful, - Active facilitation by nurse managers supported attendance.   Subtheme: Attendance Barriers   - Busy clinical work environments and staff shortages impeded attendance. | - Attendance levels improved after T2 | **Confirmation:** Quantitative results demonstrating higher attendance after T2 confirmed participants accounts that increased flexibility facilitated attendance  **Expansion:** Qualitative data revealed factors that influenced participants ability to attend. |
| Evaluate the effectiveness of the intervention on outcomes relating to nurse wellbeing | Subtheme: Opportunities for Connection and Understanding   - Widely viewed as beneficial, - Provided connection, - Opportunity to unwind and share feelings ↓stress, - Emphasised importance of self-care, - ↑ self-awareness.   Subtheme: Stressors Remain:   - Workplace stressors remained.   Subtheme: Additional Stressors:   - Emotional labour attending peer group, - Pressure to attend could ↑ stress. | - Overall mean significantly stress levels increased (p=0.04) but remained in the clinically normal range, - Mean depersonalisation scores increased (p=0.03) clinically indicating that burnout levels went from low to moderate, - Higher exposure to the intervention was associated with improved scores for depression (p=0.006), stress (p=0.004), and emotional exhaustion (p=0.02) - Participants with higher levels of attendance (exposure) to the intervention had lower levels of burnout | **Confirmation:** Quantitative results of increased stress levels and burnout confirmed qualitative findings around persistence of workplace stressors and increased challenges due to the pandemic  **Confirmation:** Stress levels remained in the clinically normal range confirming participants experiences that the intervention was beneficial  **Expansion:** Higher exposure to the intervention associated with better outcomes expanding on qualitative findings that the intervention was widely viewed as beneficial  **Discordance:** Intervention could be an additional stressor |
